# Changes in Nutritional and Techno-Functional Properties of Whole Grain Maize Flours Induced by Dry-Heat Treatment

**DOI:** 10.3390/foods13203314

**Published:** 2024-10-18

**Authors:** Marijana Simić, Valentina Nikolić, Beka Sarić, Danka Milovanović, Marija Kostadinović, Slađana Žilić

**Affiliations:** 1 Department of Food Technology and Biochemistry, Maize Research Institute “Zemun Polje”, Slobodana Bajića 1, 11000 Belgrade, Serbia; valentinas@mrizp.rs (V.N.); bsaric@mrizp.rs (B.S.); dmilovanovic@mrizp.rs (D.M.); szilic@mrizp.rs (S.Ž.); 2 Laboratory for Molecular Genetics and Physiology, Maize Research Institute “Zemun Polje”, Slobodana Bajića 1, 11000 Belgrade, Serbia; kmarija@mrizp.rs

**Keywords:** maize flour, dry heat treatment, bioactive compounds, technological properties, solvent retention capacity, in vitro digestibility

## Abstract

The present study was carried out to demonstrate the effects of dry heat treatment (DHT) at different temperatures (100, 125, 135, 150, and 165 °C) on the nutritional and techno-functional properties of white, blue, and yellow whole grain maize flour. Results showed that DHT increased the insoluble dietary fiber and free phenolic compounds of the investigated maize flours, while the bound phenolic compounds, anthocyanins, and pasting properties decreased with the rising of the applied temperature. The application of DHT caused the most notable changes regarding the amount of dietary fiber. Content of NDF (neutral detergent fiber) ranged from 11.48% to 44.35%, 14.19% to 37.84%, and 15.15% to 45.86% in white, yellow, and blue maize samples, respectively. Furthermore, at the highest temperature applied in the DHT (165 °C) the content of soluble free phenolic compounds in yellow and blue maize flour samples was 1.2- and 1.4-fold higher compared to control flour samples. DHT significantly improved the functionality of maize flour in terms of water absorption capacity, water solubility, and digestibility, thus it can be effectively used to make up for the poor functionality of raw maize flour. This study shows that DHT at moderate temperatures (125–135 °C), could be a viable solution for the pre-processing of maize flour to enhance the potential for its utilization in the food industry.

## 1. Introduction

Maize flour is commonly utilized in the preparation of a range of daily foods, such as bread, tortilla, tortilla chips, cornflakes, infant foods, and biscuits, serving as a significant source of energy for human nutrition [1]. However, due to its lack of gluten proteins, the processing of maize flour into different food products with the appropriate texture and sensory properties is a difficult task. Several authors reported dry and wet heat treatments as alternative ways of grains and flours modifications, including heating, roasting, steaming, and extruding [2,3,4,5]. This implies that it is necessary to modify the proteins and starch of gluten-free cereals to obtain textural properties that are similar to the proteins and starch in wheat flour.

Dry-heat treatment (DHT) is a safe physical-modification methods that typically involves heating at temperatures of 110 to 150 °C for 1 to 4 h while maintaining a low moisture content (<10% *w*/*w*) [6]. DHT is a commonly used method for processing maize flour in many countries. Starch gelatinization and protein denaturation are, from a technological perspective, the most significant modifications that take place during heat treatments. The starch granules can sustain damage caused by DHT, which lead to changes in the crystalline and amorphous portions of starch’s structure [7]. Consequently, starch granules form aggregates with smaller diameters than those found in native flours [8]. Furthermore, Oh et al. [6] found that after being dry heated at 110, 130, and 150 °C for 1, 2, and 4 h, the crystalline structure type of rice starch did not change, but the peak X-ray diffraction intensities of the dry heated starch decreased as the heating period increased. Marston et al. [9] evaluated the functional effects of 95 °C and 125 °C DHT on sorghum flour, and observed promising results related to the volume and sensory properties of breads and cakes. These effects are due to the modified gelatinization properties of starch and its increased ability to absorb water. Additionally, the DHT can alter the physicochemical properties of starch, such as its viscosity, swelling power, and solubility, and enhance its functional properties. Sun et al. [10] found that millet starch’s peak viscosity decreased after being dry-heated at 130 °C for 2 or 4 h, whereas the trough and final viscosities increased. Additionally, the use of heat-treated maize flour enhances the dough and bread yield, delays aging, and extends the shelf life of bakery products due to its strong ability to bind and retain water [11,12]. According to research by Giordano et al. [13], DHT could be beneficial for reducing lipase activity and, consequently, increasing the storage stability suitable for food uses. Besides, heat treatment affected the physicochemical properties of proteins, enabling molecules to assemble into larger aggregates and changing their shape and molecular size by rupturing existing bonds and generating new ones [5]. During heat treatment, proteins denature and become more digestible, as their secondary and tertiary structures are altered. However, changes to the side chains of amino acids can slow down the action of some digestive enzymes, and the creation of links inside or between molecules could decrease the protein molecule’s overall digestibility [8].

The positive effects of heat-treated gluten-free flours on the quality of flour-based products have been increasingly recognized. However, to the best of our knowledge, no studies have been performed on the dry heating of maize flour. Only a limited amount of research has been previously conducted on the impact of DHT on maize flour, whereas moist heat treatments, such as steaming and extrusion, have been explored to a deeper extent in the scientific literature. Taking the above-mentioned facts into account, the main aim of the present research was to determine the effects of DHT on the physicochemical properties and changes in the rheological parameters of the investigated maize flours. To achieve this goal, the chemical composition of maize flour samples was determined, as was the percentage of damaged starch, the content of bioactive compounds, and the antioxidant capacity. The secondary objective of this investigation was to determine whether DHT applied at various temperatures affects flour’s in vitro digestibility, gelling, and pasting properties, along with SRC (solvent retention capacity).

## 2. Materials and Methods

### 2.1. Plant Material

The plant material encompassed three different maize hybrids (*Zea mays* L.) recently developed at the Maize Research Institute Zemun Polje, Serbia (44°52′ N, 20°19′ E, 82 m a.s.l.), sown in the 2022 growing season. The genotypes were chosen on the basis of kernel color (white, blue, and yellow), as well as their differences in kernel hardness (white dent maize-*Zea mays* var. *identata*; blue and yellow popping maize- *Zea mays* var. *everta*), and agronomic traits such as yield and its components. To maintain the plots free of weeds and diseases and to ensure sufficient nutrition, standard cropping methods were used. Kernels of white, blue, and yellow maize were ground in a laboratory mill (Perten 120, Perten Instruments, Hägersten, Sweden) to a powder (particle size < 500 μm), without removing the germs before milling.

### 2.2. Dry-Heat Treatment of Maize Flour Samples

Maize flour samples (70 g) were evenly thinly spread in a Petri dish (ϕ185 mm) and thermally treated under dry conditions in a ventilation oven (Memmert UF55, Memmert GmbH^+^Co. KG, Schwabach, Germany) for 60 min at 100 °C, 125 °C, 135 °C, 150 °C, and 165 °C. The fan and flap were set to 0% and 20%, respectively. The choice of treatment temperatures was based on the temperature range commonly used in different heat-based treatments reported in the literature as well as in the food industry. Flour samples with no temperature treatment were considered to be the control samples. The flour samples were allowed to cool down at room temperature (~20 °C) and were stored in hermetic bags until required for analyzes. Three batches were carried out for each treatment temperature for each maize genotype.

### 2.3. Chemical Procedure

#### 2.3.1. Analysis of Total Protein Content in Maize Flour Samples

The protein content was determined by the standard micro-Kjeldahl method [14]. A total of 0.2 g of ground maize flour sample was weighed into a tube and mixed with concentrated H_2_SO_4,_ and with a catalyst, and heated for a period of 45 min at 420 °C in a BÜCHI Kjeldahl System (Speed Digester K-439, BÜCHI Labortechnik, Flawil, Switzerland). After cooling, the tube was placed in a BÜCHI Kjeldahl System (Auto Kjeldahl Distilation Unit K-350). Nitrogen present in the digested sample was then neutralized by treating it with a sodium hydroxide (NaOH) solution, releasing ammonia gas (NH_3_) from ammonium sulfate ((NH_4_)_2_SO_4_). The distillate collected in a receiving flask with extra boric acid (H_3_BO_3_) was titrated using a standard acid and an appropriate end-point indicator to determine the sample’s overall nitrogen content. The protein content was expressed as the total N multiplied by 6.25. The results are expressed in percentages per dry matter (d.m).

#### 2.3.2. Analysis of Starch Content in Maize Flour Samples

The Ewers method [15] was used to determine the starch content on a polarimeter (UniPol L 2020, Schmidt + Haensch GmbH & Co., Berlin, Germany). All the results are given as the percent per d.m.

#### 2.3.3. Analysis of Dietary Fibre Content in Maize Flour Samples

The Van Soest detergent method, modified by Mertens [16], was used to measure the levels of hemicellulose, cellulose, neutral detergent fibers (NDF), acid detergent fibers (ADF), and acid detergent lignin (ADL) using the Fibertec system FOSS 2010 Hot Ex-tractor (FOSS Tecator, Hoeganaes, Sweden). The technique is predicated on the fibers’ solubility in chemicals that are neutral, acidic, and alkaline. In basic terms, NDF stands for total insoluble fibers (those that are not soluble in water), whereas ADF mostly comprises cellulose and lignin, and ADL is composed of pure lignin. After sifting and boiling the sample (1 g) for 60 min at a pH 7 neutral solution in 100 mL of a specific detergent, the NDF were determined. The liquid that went through the sintered disc filter included dissolved substances, such as protein, sugar, and starch. The determination of ADF was essentially the same, with the exception that acid (pH 2) conditions required the use of a different detergent. The varying detergent and acid conditions caused the hemicellulose and cell solubles to dissolve and be filtered out. After subjecting ADF to a stronger acid (72% H_2_SO_4_) to breakdown cellulose, the ADL was determined. The NDF, ADF, and ADL have been estimated as a percentage of the initial sample following filtration and drying. The difference between the NDF and ADF contents was used to determine the hemicellulose content, and the difference between the ADF and lignin contents was used to calculate the cellulose content. Each result is presented as a percentage per d.m.

#### 2.3.4. Extraction of Free and Bound Phenolic Compounds from Maize Flour Samples

Extracts of soluble phenolics were obtained by vigorously shaking 0.5 g of sample in 20 mL of 7:7:6 (methanol:acetone:H_2_O) for 30 min at room temperature. For the detection of free soluble phenolic compounds, 5 mL of extracts were evaporated under a N_2_ stream at 30 °C to dryness (Reacti-Therm nitrogen evaporator system 18821, Thermo Fisher Scientific Inc., Waltham, MA, USA), and final residues were redissolved in methanol.

Extracts of bound phenolics were obtained by vigorously shaking 0.5 g of sample in 10 mL of 4 M NaOH for 4 h at room temperature. After alkaline hydrolysis, the pH was adjusted to 2.0 (6 N HCl), and extraction was performed with ethyl acetate and diethylether (1:1, *v*/*v*) four times. For the detection of bound insoluble phenolic compounds, 5 mL of combined extracts were evaporated under the N_2_ stream at 30 °C to dryness, and final residues were redissolved in methanol.

#### 2.3.5. Analysis of Total Phenolic Content in Maize Flour Samples (TPC)

The concentration of total phenolic compounds in the MeOH extracts was determined spectrometrically using the Folin–Ciocalteu method [17], using gallic acid as a standard to prepare a calibration curve. The extract was transferred into a test tube, and the volume was adjusted to 500 μL with distilled water and mixed with 250 μL of Folin–Ciocalteu reagent. After 5 min, the mixture was neutralized with 1.25 mL of 20% aqueous sodium carbonate solution, and after 40 min, the absorption of the reaction mixture was measured at 725 nm against a methanol blank using an Agilent UV/vis spectrophotometer (Agilent Technologies, Santa Clara, CA, USA). The results were expressed as milligrams of gallic acid equivalent (GAE) per gram of d.m.

#### 2.3.6. Analysis of Total Anthocyanin Content in Maize Flour Samples (TAC)

Extracts of anthocyanins were obtained by vigorously shaking 50 mg of flour sample in 5 mL of MeOH acidified with 1 M HCl (85:15, *v*/*v*) for 30 min in a horizontal shaker (MLW Thys 2, VEB MLW Labortechnik, Ilmenau, Germany). After shaking the absorbance was measured on a spectrophotometer at 535 and 700 nm [18]. The content was calculated using the molar extinction coefficient of 25.965 Abs/M·cm and a molecular weight of 449.2 g/mol and expressed as mg of cyanidin 3-glucoside equivalent (CGE) per kg of d.m.

#### 2.3.7. Analysis of the Total Antioxidant Capacity of Maize Flour Samples

The total antioxidant capacity was measured according to the direct or QUENCHER method described by Serpen et al. [19] using the ABTS (2,2′-azino-bis (3-ethilbenzothia-zoline-6-sulfonic acid)) reagent. A stock solution of ABTS^•+^ was prepared by mixing a 7 mM aqueous solution of ABTS^•+^ with 2.45 mM K_2_O_8_S_2_ (final concentration) and allowing the mixture to stand in the dark at room temperature for 12–16 h before use. On the day of analysis, an ABTS^•+^ working solution was obtained by diluting the stock solution in water/ethanol (50:50, *v*/*v*) to overcome the solubility-dependent low reactivity of antioxidants in a solid sample toward ABTS^•+^. The absorbance of the ABTS^•+^ working solution was 0.70 ± 0.02 AU at 734 nm. Maize flour (10 mg—white and yellow and 5 mg—blue) was mixed by adding 20 mL of ABTS^•+^ working solutions, and the mixture was rigorously shaken for 30 min. After centrifugation at 10,000× *g* for 5 min, the optically clear supernatant was separated, and the absorbance measurement was performed at 734 nm. The total antioxidant capacity was expressed as mmol Trolox equivalents (Eq) per kg of d.m.

### 2.4. Solvent Retention Capacity (SRC)

According to the American Association of Cereal Chemists method 56-11 adapted by Haynes et al. [20], four solvents were individually used to determine the SRC values: water; 50% sucrose in water; 5% sodium carbonate in water; 5% lactic acid in water. In centrifuge tubes (50 mL), 5 g of flour and 25 mL of an appropriate solvent were added. The mixture was vortexed vigorously for 5 s to suspend the flour. The samples were vortexed for 20 min at 1 min intervals after every 5 min to allow the samples to solvate and swell. The centrifugation was performed at 3000 rpm for 10 min. The supernatant was discarded, and the wet pellet that was obtained was allowed to decant for 10 min before it was weighed. The SRC values were reported as the percentage of the weight of flour gel after exposure to the solvent divided by the original flour weight.

### 2.5. Gelling Properties of Maize Flour Samples

The water solubility index (WSI), water absorption index (WAI), and the swelling power (SP) were determined according to the method described by Cornejo and Rosell [21]. The samples (1 g) were weighed into previously tared centrifuge tubes and 20 mL of distilled water was added. The tubes were shaken in a water bath for 15 min at 90 °C and then centrifuged at 3000× *g* at 4 °C for 10 min. The supernatants were carefully removed into Petri dishes and evaporated in a ventilation oven for 12 h at 110 °C. Each sample was analyzed in three replicates, and WSI, WAI, and SP were calculated using the following equations:

(1) $$\mathrm{WAI}\;\left(\frac{g}{g}\right)=\frac{Weight\;of\;sediment}{Sample\;weight}$$

(2) $$\mathrm{WSI}\;\left(\frac{g}{g}\right)=\frac{Weight\;of\;dissolved\;solids\;in\;supernatant}{Sample\;weight}$$

(3) $$\mathrm{SP}\;\left(\frac{g}{g}\right)=\frac{Weight\;of\;the\;sediment}{\left(Sample\;weight-Weight\;of\;dissolved\;solids\;in\;supernatant\right)}$$

### 2.6. Analysis of Starch Damage Level in Maize Flour Samples

The starch damage level in samples was determined by using a Megazyme Kit (Megazyme Wicklow, Ireland) K-SDAM, and following the official AACC method number 76-31.01. [22].

### 2.7. Analysis of Pasting Properties of Maize Flour Samples

Changes in the apparent viscosity of aqueous suspensions (8% starch suspension, total mass of 500 g) were analyzed in order to obtain the pasting curves of the investigated maize flour samples. Suspensions were heated in a viscograph from 25 to 95 °C at a rate of 1.5 °C/min. The suspensions were thermostated at 95 °C for 30 min, cooled to 50 °C, and kept for another 10 min. The Brabender Viscograph (model PT 100, C.W. Brabender Instruments, Inc., Duisburg, Germany) was operated according to the official methods and the viscosities were expressed in Brabender units (BU) [23].

### 2.8. In Vitro Multistep Enzymatic Digestion Protocol

A multistep in vitro protocol was followed to determine the digestibility of whole-grain maize flour. The oral, gastric, duodenal, and colon phases of the foods’ gastrointestinal digestion are included in a procedure that was first reported by Papillo et al. [24] and then altered by Hamzalıoğlu and Gökmen [25]. The method was carried out without aiming to fully replicate the digestion processes.

Digestion fluids representing saliva (simulated salivary fluid, SSF), gastric juice (simulated gastric fluid, SGF), and duodenal juice (simulated duodenal fluid, SDF) were employed to recreate the conditions found in the human gastrointestinal tract. The preparation of these digestive fluids is indicated in Table 1.

Five milliliters of SSF were added to a glass flask with a screw top after five grams of whole grain wheat flour had been added. For two minutes, the flaps were vortexed to mimic the oral passage. Following the addition of 10 mL of SGF and 5 mL of pepsin solution (12.5 mg/mL in 0.1 M HCl), the mixture’s pH was brought to 2.0. After that, to replicate the gastric phase, the acidified mixture was shaken for two hours at a pace of sixty strokes per minute at 37 °C. The SDF solution was used to dissolve the bile salts to a 10 mg/mL concentration. The gastric phase was followed by a pH adjustment to 7.5. Next, 5 mL of pancreatin solution (10 mg/mL in distilled water) and 20 mL of the SDF with bile salts mixture were added to the flask. The process involved incubation at 37 °C and continuous shaking for two hours at a rate of 60 strokes per minute to replicate the duodenum phase. Then, 5 mL of a 1 mg/mL, pH 8.0 protease solution were added, and the mixture was shaken for one hour at 37 °C to replicate the colon phase. Finally, 150 µL of Viscozyme L was added, and the mixture was shaken for 16 h at a speed of 30 strokes per minute at 37 °C.

Following in vitro digestion, samples were passed through quality filter paper and dried for four hours at 105 °C to maintain mass. Following sample weighing, the digestibility was determined using the following formula:

Digestibility = ((m_0_ − m_d_)/m_0_)·100 (4)

where m_0_ is the mass of absolutely dry sample before digestion and m_d_ is the remaining (undigested) mass of absolutely dry sample.

### 2.9. Measurement of Colour of Maize Flour Samples

The color properties of maize flour samples were measured instrumentally by using Konica Minolta colorimeter (CR-400/410, Konica, Minolta, Tokyo, Japan), which was calibrated against a white calibration standard (CM-A70). The surface colors of flour samples were given as average L*—brightness (from 0 (black) to 100 (white)), a*—greenness/redness (from a* < 0 (green) to a* > 0 (red)), b*—blueness/yellowness (from b* < 0 (blue) to b* > (yellow)) values of four measurements per sample.

### 2.10. Statistical Analysis

The data are given as the average ± standard deviation of a minimum of three independent replications. Version 5.0 of the Statistica program (StatSoft Co., Tulsa, OK, USA) was used to statistically analyze the data. Using the Tukey’s test, the significance of the differences between the samples was examined. Differences between the means with probability *p* < 0.05 were considered statistically significant. Inter-relations were analyzed using the Statistica software Minitab^®^ version 16.1.0 (Minitab, LLC, State College, PA, USA).

## 3. Results and Discussion

### 3.1. Chemical Composition of Maize Flour Samples

The chemical composition of the investigated maize flour samples is presented in Table 2. In order to evaluate the impact of DHT on the chemical composition of maize flour samples, all the results were compared with those of non-treated maize flour samples as a control. The protein content in the control samples of the tested maize flour had the following decreasing order: blue maize > yellow maize > white maize. According to our results, the protein content in maize flour samples ranged from 8.24% to 8.68%, 11.97% to 12.39%, and 12.83% to 13.05% in white, yellow, and blue flour samples, respectively. The total protein content in our study was not significantly influenced by DHT. Although the protein content of the control and treated samples were quite similar, we observed some significant differences after the application of DHT. Maize flour samples treated at higher temperatures (150 °C and 165 °C) had lower protein contents than the untreated samples, which could be related to some changes brought on by the heating procedure and the mechanical energy used. According to Félix-Medina et al. [26], Téllez-Morales et al. [27], Jebalia et al. [28], and Ramos-Díaz et al. [29], these modifications could be protein cross-linking, disulfide bonds formation or the development of Maillard reaction products, and all of them may limit the nitrogen determination by Kjeldahl method and thus, reduced protein values in thermally treated samples. Our results are consistent with those of Rolandelli et al. [4] who reported quite similar protein content in the control and heat-treated maize flour, as well as those of Torbica et al. [8], who found that the total protein content in barley and triticale were not significantly affected by DHT, while in rye, oat, sorghum, and millet this treatment lowered protein content. However, there are limited reports on the structural and physicochemical alterations caused by heat in maize (alcohol-soluble) proteins.

Sun et al. [30] reported that heat treatment induced significant changes of physical, structural, thermal, and morphological characteristics of maize proteins. These results demonstrated that heat treatment caused intermolecular interactions between zein molecules, which changed the configuration of the protein. Furthermore, taking into account the importance of proteins in food products and their co-existence with starch in many foods, studying the effects of DHT on starch is also of great importance. Results of total starch content (Table 2) point out that the control white maize flour sample represents the richest source of starch compared with control samples of yellow and blue maize flour. According to our results, the starch content ranged from 70.08% to 74.45%, 68.37% to 69.61%, and 64.63% to 67.33% in white, yellow, and blue maize flour samples, respectively. In all investigated maize flour samples, the highest starch content was determined at a temperature of 150 °C. The DHT applied at lower temperatures, 100 °C and 125 °C, slightly decreased the starch level. In this investigation, there was no discernible trend in how DHT affected the starch content, which suggested that DHT has a more significant effect on the structural properties of flour than individual constituents such as starches. These observations are consistent with those of Qiu et al. [31] and Sun et al. [10]. Furthermore, Oh et al. [6] and Vashisht et al. [32] reported that DHT may cause starch molecules to reassociate and starch crystallites to reorganize, which could improve the consistency of starch crystallization. However, DHT also has some disadvantages. According to Li et al. [33] starch granules may be slightly harmed, which could lead to a reduction in the physicochemical qualities of starch. Correspondingly, the structural change of crystalline and amorphous regions of starch and the destruction of intermolecular hydrogen bonds, as well as the enhancement of the bonding between starch chains and the bonding force within granules and thus resulting in the reduction in the pasting viscosity.

The changes that occurred as a result of the application of DHT were most pronounced in terms of dietary fiber content. The content of NDF ranged from 11.48% to 44.35%, 14.19% to 37.84%, and 15.15% to 45.86% in white, yellow, and blue maize samples, respectively. According to the results presented in Table 2, the highest content of NDF was detected at a temperature of 165 °C in all tested maize flour samples. The NDF content at this temperature was 3.9-, 2.7- and 2.9-fold higher than that found in the control samples of white, yellow, and blue maize flour samples, respectively. Furthermore, the results indicate that the increase in the NDF concentration was moderate with no significant differences up to a temperature of 135 °C; however it significantly rose afterward. Thus, at a temperature of 135 °C, the content of NDF in the blue maize flour sample was 50% higher and amounted to 29.98%. At the same temperature, the NDF concentration increased by 1.4- and 1.3-fold in the samples of white and yellow maize flour, respectively. The content of ADF ranged from 1.98% to 4.41% and from 3.51% to 5.33% in yellow and blue maize flour samples, respectively. Furthermore, the content of ADF in the control sample of white maize flour amounted to 3.56% which was about 11.4% lower than that found in the DHT sample at a temperature of 165 °C. The same trend was observed in the DHT samples of yellow and blue maize flour at 165 °C, with 18.82% and 29.27% higher content of ADF compared to the control samples, respectively. Statistically significant differences in the ADF content were not observed between samples thermally treated between 100 °C and 150 °C. In addition, our results for ADL content showed the most pronounced effect of DHT at a temperature of 165 °C. The content of ADL in the control sample of white, yellow, and blue maize flours amounted to 0.45%, 0.40%, and 0.54%, respectively. DHT caused an intensive increase in ADL content at a temperature of 165 °C and it was 72.2%, 64.6%, and 76.4% higher than that found in the control samples of white, yellow, and blue maize flour, respectively. These results demonstrate that the heat treatment’s impact on dietary fiber depends on the type of fiber used and the conditions under which it is processed. In support of this, the results of Torbica et al. [8] stated that DHT caused a more intensive decrease in insoluble dietary fiber than extrusion in different grain flours, while heat treatments of total dietary fiber had no statistically significant impact. Additionally, research by Chang [34] reported that treatment of autoclaving tended to decrease the total dietary fiber while microwave heat tended to increase the total dietary fiber in corn fiber. Cellulose and hemicellulose, which are principal non-starch polysaccharides present in maize grain [35] ranged from 1.73% to 3.28% and from 7.92% to 40.47%, respectively. Results showed that DHT has the same effect on hemicellulose content as previously on the NDF content, causing a minor increase in hemicellulose concentration without significant differences up to a temperature of 135 °C, but then a considerable increase. The hemicellulose content at the highest DHT temperature was 5.1-, 3.2-, and 3.42-fold higher than that found in the control samples of white, yellow, and blue maize flour samples, respectively.

The obtained results revealed that DHT had great effects on the physicochemical properties of maize flour constituent structural reorganization which could impact the functional and technological properties of the flour.

### 3.2. Bioactive Compounds of Maize Flour Samples

Contents of phenolic compounds and anthocyanins of control and thermally treated maize flour samples are given in Table 3. There were noticeable variations in the content of phenolic compounds among all tested maize flour samples (Table 3). The control sample of blue maize flour had a higher soluble free phenolic content (390.21 mg GAE/kg d.m.) than those of white and yellow maize flour (145.89 and 300.83 mg GAE/kg d.m., respectively). These results were expected, since numerous investigations reported that blue maize is especially high in phenolic compounds as compared to light-colored maize genotypes [36,37,38].

According to our results, the DHT increased the content of soluble free phenolic compounds in tested maize flour samples in comparison to those in control maize flours. However, at a temperature of 100 °C, a reduction of 10%, 11.3%, and 13.5% was observed for white, yellow, and blue maize flour samples, respectively. DHT caused a more significant increase in soluble free phenolic compounds in white maize flour than in yellow and blue flour samples. The content of soluble free phenolic compounds in white flour treated at a temperature of 165 °C was 130.6% higher than that in the control sample. These increases may be partially attributed to the release of bound phenolic acids during DHT, but they may also result from the Folin–Ciocalteu reagent’s ability to react with any reducing agent when used in analysis of phenolic compounds. This result is in accordance with the previous research of Žilić et al. [39] who stated that the Maillard reaction products, which have reductone structures, contributed to the increase in total phenolic compounds in cookies measured by the Folin–Ciocalteu assay. Furthermore, at the highest temperature applied in the DHT (165 °C) content of soluble free phenolic compounds in yellow and blue maize flour samples was 1.2- and 1.4-fold higher compared to control flour samples. These differences suggest that even among a small group of maize hybrids, there are genotypic variations for phenolic compounds as well as their physiochemical properties during thermal processing. Unlike the soluble free phenolics, insoluble bound phenolics showed a significant decrease with the treatment temperature rising, and the lowest values were obtained at 125 °C. These reductions in white, yellow, and blue maize flour samples amounted to 17.8, 12.5, and 30.4%, respectively. This could be explained by the fact that bound forms of phenolic compounds are released during thermal treatments due to the breakdown of the cell matrix, which can lead to the release of phenolic compounds from their bound state into their free state. These results are in accordance with the literature data [40,41,42].

As we expected, in complete contrast to white and yellow maize flour samples, the blue maize flour samples were rich in anthocyanins (894.62 mg CGE/kg). According to Simić et al. [43], variations in the total anthocyanin concentration can be mainly ascribed to the anthocyanins’ position inside of the maize grain, which in turn affects their variable availability. In this study, DHT exerted both positive and negative effects on anthocyanin content. DHT showed a significant increase in the anthocyanin content of blue maize flour samples up to a temperature of 135 °C, while at higher temperatures intense degradation of anthocyanins was observed. The content of anthocyanins was reduced by 21% and 59% in the flour heated at 150 °C and 165 °C, respectively. This is in agreement with results reported by Blanch and Ruiz del Castillo [44], who reported a reduction in anthocyanin content in the whole-grain black maize flour heated at 150 °C, 180 °C, and 200 °C. Given that anthocyanins are the main antioxidants in colored maize grains [44], the obtained highest antioxidant capacity in blue maize flour was as expected. The antioxidant capacity of the control blue maize flour sample amounted to 24.03 mmol trolox/kg, which was about 2- and 1.4-fold higher than that found in white and yellow maize flour samples, respectively. However, De la Parra et al. [45] did not find significant differences in antioxidant capacity between blue and white maize tortillas. The antioxidant capacity ranged from 10.05 to 13.32, 14.83 to 17.15, and 20.51 to 24.03 mmol trolox/kg in white, yellow, and blue maize flour samples, respectively. Changes in the antioxidant capacity of tested maize flour samples in relation to DHT are presented in Table 3, and it is evident that DHT did not show a clear trend in its effect on antioxidant activity. However, Li et al. [46] assessed how heat treatment affected the bioactive compounds and antioxidant capacity of heat-treated and untreated purple wheat bran and the muffins made from it. According to the findings, heat treatment under selected conditions did not significantly alter the antioxidant activity of purple wheat bran.

### 3.3. Techno-Functional Properties of Maize Flour Samples

**Figure 1:**
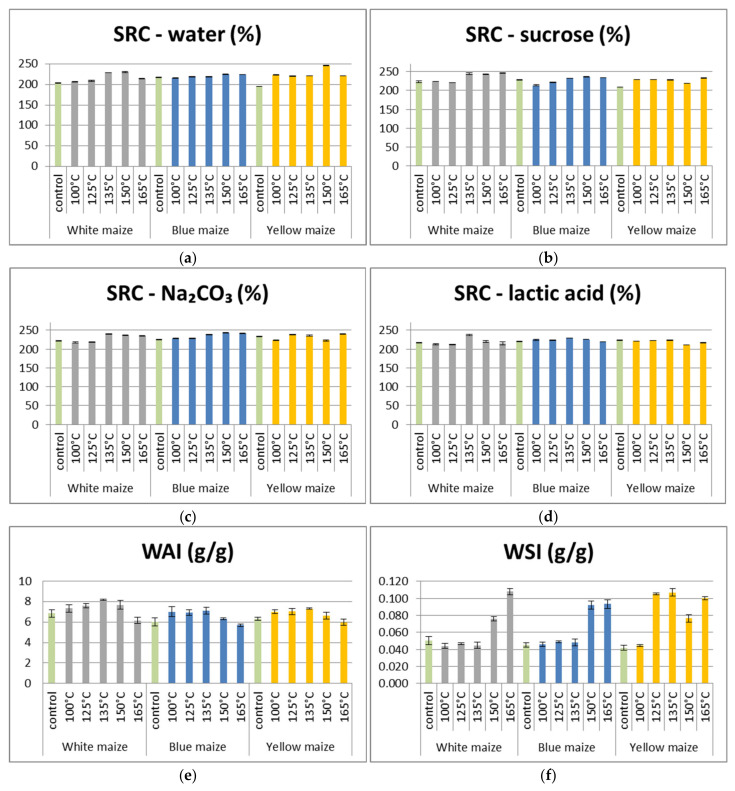

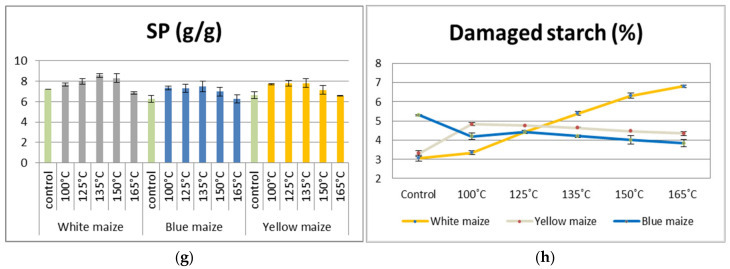
Solvent retention capacity, gelling properties and damaged starch of maize flour samples: (**a**) SRC of water; (**b**) SRC of sucrose; (**c**) SRC of water; (**d**) SRC of water; (**e**) water absorption index; (**f**) water solubility index; (**g**) swelling power; (**h**) damaged starch.

**Figure 2:**
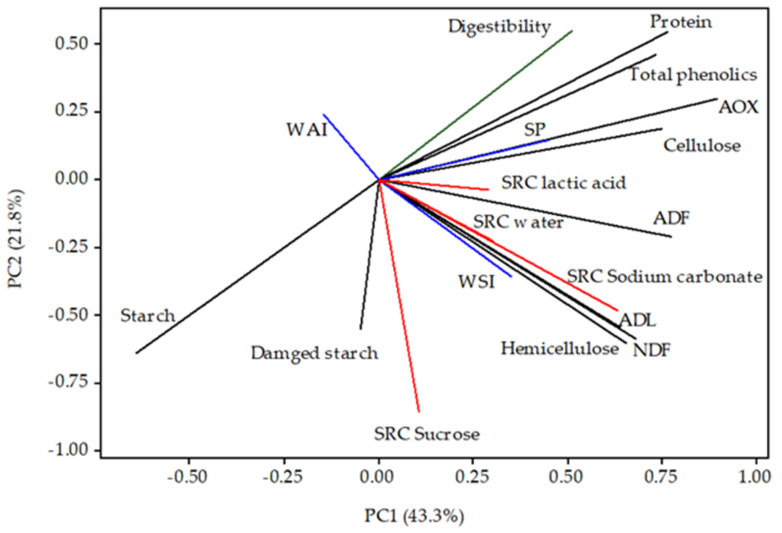
Inter-relations of solvent retention capacity (SRC-water, sucrose, lactic acid and sodium carbonate), gelling properties (WAI, WSI, and SP), damaged starch and in vitro digestibility with chemical compounds of DHT maize flour samples.

Given the established correlation between rheological and baking quality-related parameters and solvent retention capacity (SRC) [47], it is important to investigate the effects of DHT on these properties. SRC of maize flour samples are presented in Figure 1a–d. The ability of the control and DHT maize flour samples to retain the tested solvents varied significantly (*p* < 0.05), as Figure 1 illustrates. The obtained results demonstrated that the application of DHT considerably altered the SRC properties of the maize flour samples. At temperatures over 135 °C, DHT significantly enhanced the samples of white and blue maize flour’s ability to retain sucrose. This was expected since the sucrose retention capacity positively correlates with the properties of dietary fiber (Figure 2), and in all of the studied maize flour samples, the highest content of dietary fibers was found at the highest temperature of DHT. Furthermore, the highest retention capacity of the lactic acid was detected in all DHT maize flour at a temperature of 135 °C. Given that the SRC of lactic acid is related to the properties of proteins in flour samples (Figure 2), and no statistically significant difference was observed in the protein content (Table 2) at different DHTs, there were obviously structural changes due to protein denaturation that enhanced the binding sites available for lactic acid that we did not detect by standard methods of protein analysis.

DHT did not manifest a clear trend in its effect on sodium carbonate SRC. It was expected that white maize flour samples have a higher ability to retain sodium carbonate at higher temperatures of DHT since the sodium carbonate SRC is correlated with the levels and swelling of damaged starch in flour [48,49] (Figure 2). But while the amount of damaged starch in the white maize samples rose as the DHT’s temperature rose, this did not contribute to the rise in sodium carbonate SRC. These decreases may be partially attributed to the gelatinization process of starch granules during DHT, which may affect their ability to interact with sodium carbonate. Therefore, these results suggest that the relationship between damaged starch and sodium carbonate SRC is more complex than initially anticipated.

According to our results, the damaged starch content ranged from 3.06% to 6.80%, 3.29% to 4.34%, and 5.30% to 3.84% in white, yellow, and blue maize flour samples, respectively (Figure 1h). The lowest content of damaged starch in control samples of white maize flour was expected since the dent maize kernel type has a lower resistance to milling compared to popping maize kernels. These results are in line with those of earlier reports [50]. Results presented in Figure 1h showed that the DHT significantly influenced the content of damaged starch in maize flour samples. DHT caused an intensive increase in damaged starch content at a temperature of 165 °C, and it was 2.2-fold higher than that found in the control sample of white maize flour. In complete contrast to white maize flour samples, the blue maize flour samples record a 1.4-fold decrease in damaged starch content at the same temperature. Nevertheless, it should be noted that various technological properties benefit from moderate levels of damaged starch since it increases fermentation activity and improves hydration [51], while excessively damaged starch can cause a large quantity of water to be absorbed, which can speed up the enzymatic process and cause sticky dough and lower product volume [52]. The inter-relation presented in Figure 2 confirmed previous investigations, since it demonstrated positive correlation between damaged starch content and WSI that represents the measure of the hydration properties of the maize flour. Taking all of the above-mentioned facts into account, DHT at a temperature of 125 °C with a moderate amount of damaged starch in all maize flour samples, could have a favorable effect on many techno-functional properties of maize products.

The water absorption index (WAI), water solubility index (WSI), and swelling power (SP) of maize flour samples are presented in Figure 1e–g. The obtained results showed that the gelling properties of the flour changed significantly with the applied DHT. Thus, the WAI ranged from 6.152 to 8.174, 5.983 to 7.306, and 5.684 to 7.112 g/g in white, yellow, and blue maize flour samples, respectively (Figure 1). According to the results presented in Figure 1, the highest WAI was detected at a temperature of 135 °C in all tested maize flour samples. Compared to their control samples, the obtained values of WAI in the white, yellow, and blue maize flour samples thermally treated at 135 °C were higher by 16%, 14%, and 15%, respectively. Moreover, the results indicate that the WAI significantly increases up to a temperature of 135 °C, but afterward, it significantly decreases. These findings agree with those of Patil et al. [53], who reported that milder heat process conditions were associated with a greater improvement in WAI. Our results indicate that the particular DHT temperatures have a greater impact on the structural characteristics of starch granules’ conditioning to absorb more water and swell for the desired consistency in the end product, which enhances yield and consistency and gives the food a more distinct texture. Furthermore, Osundahunsi [54] states that WSI is used to indicate the starch degradation level and reflect the quantity of soluble polysaccharides that are released from the starch granules and discharged into the aqueous phase. However, our results (Figure 2) demonstrated no correlation between WSI and starch. DHT at a lower temperature (under 150 °C) produced the lower WSI with a range from 0.044 to 0.047, and from 0.046 to 0.049 g/g in white and blue maize samples, respectively. These lower WSI values suggest that WSI may include opposing effects, like molecular interactions within degraded components (lipids, proteins, and carbohydrates) at the molecular level, which raises molecular weight and decreases solubility, thus lowering WSI [55]. The WSI value at temperature of 165 °C was 2.2-, 2.4-, and 2.1-fold higher than that found in the control samples of white, yellow, and blue maize flour samples, respectively. The white maize sample had the highest WAI and lowest WSI when treated with DHT at 135 °C, which suggests that white maize flour swells more easily and could exhibit a higher viscosity. Our results are consistent with those reported for barley, rye, triticale, oats, sorghum, and millet [8]. According to Bryant et al. [56], flour with a higher WAI and a lower WSI could be utilized in a product where a high viscosity is the main concern.

The effect of DHT on the SP of tested maize flour samples is presented in Figure 1. For all maize samples, the SP significantly increased with rises in treatment temperature, and the highest value was obtained at 135 °C. Similarly to WAI, the SP remarkably decreases at temperatures higher than 135 °C. The SP ranged from 6.854 to 8.565, 6.565 to 7.801, and 6.270 to 7.470 g/g in white, yellow, and blue maize flour samples, respectively. A gradual decrease in the SP of starch could be related to an increase in the amylose–amylopectin interaction due to the rearrangement of starch molecules under DHT [57]. Our results demonstrated the same correlation (Figure 2), while the study of Sun et al. [10] has recorded that the heat moisture-treated sorghum flour showed a significant decrease in swelling capacity due to the structural rearrangement and re-associations of starch polymer amylose–lipid complexes formation within starch granules.

### 3.4. Pasting Properties of Maize Flour Samples

**Figure 3:**
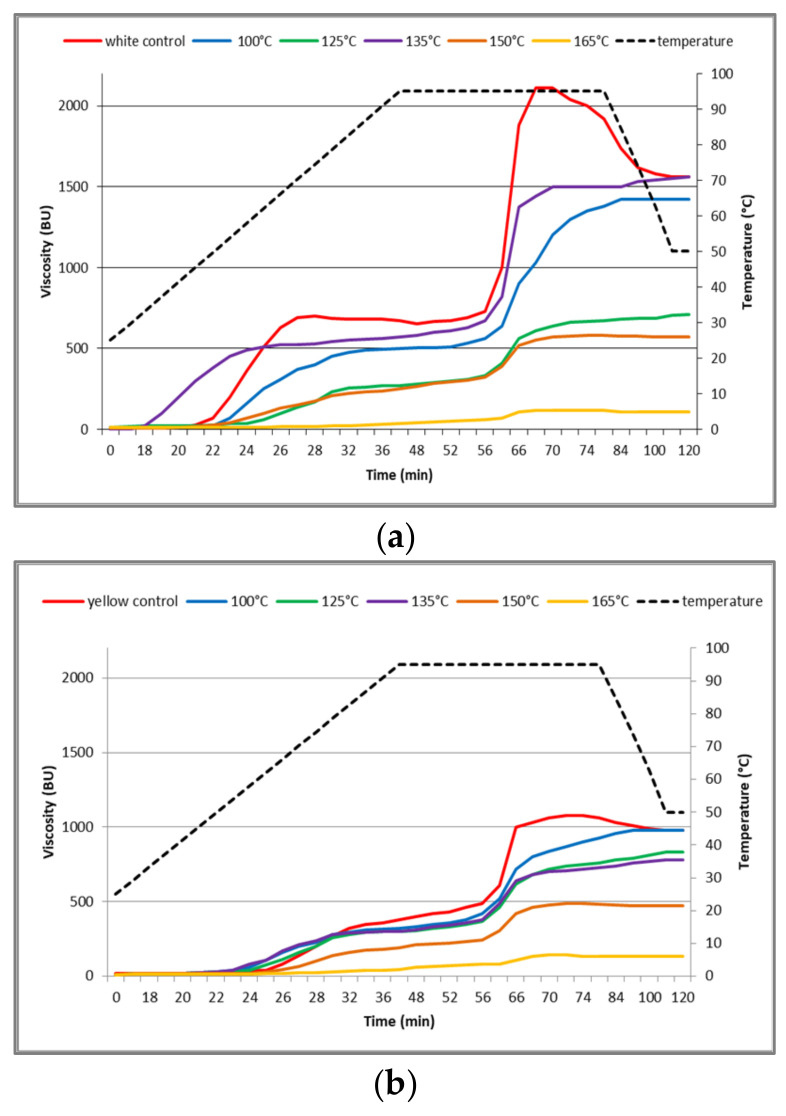

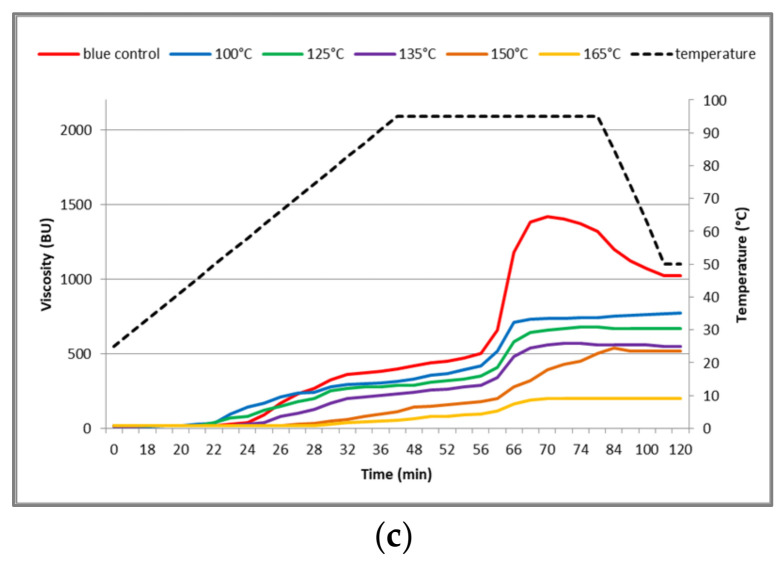
Pasting profiles (viscosity curves) of investigated maize flour samples: (**a**) white maize, (**b**) yellow maize, and (**c**) blue maize.

The pasting properties of control and thermally treated maize flour samples are presented in Figure 3. According to our results, the DHT affected the pasting properties of maize flour samples quite similarly. With the increase in the DHT temperature, the peak viscosity, final viscosity, and setback viscosity of maize flour samples were all significantly decreased, which suggest that there was a negative correlation between the viscosity and the temperature of the DHT. The decrease in viscosity could also have been caused by the breakdown of the starch granules followed by the linear orientation of the polymer [58]. Besides, the breakdown viscosity of treated maize flour samples showed a decreasing trend, which implies that DHT-affected starch becomes more resilient to thermal and mechanical shear. Hence, our maize flour samples are not suitable for use in products that require a higher final viscosity and hot paste stability. Similar results were reported for maize starch [59]. Findings reported by Sun et al. [10] showed that DHT on millet flour significantly changed the flour functionality and increased pasting viscosities. Peak viscosities of the control white, yellow, and blue maize flour samples amounted to 700 BU, 350 BU, and 360 BU, respectively. As expected, results indicate that differences in the genetic background of the maize hybrids affected the differences in the properties of starch gelatinization. Our results are well in accordance with research on proso millet starch reported by Mahajan et al. [60]. In addition, the white maize flour sample’s pasting curve at 135 °C showed the smallest decrease in maximum viscosity and the final viscosity was equal when compared to the control sample. These results validate the findings of our conclusion on gelling properties, which show that molecular interactions within broken components increase molecular weight and decrease solubility, hence reducing WSI.

### 3.5. In Vitro Digestibility

**Figure 4:**
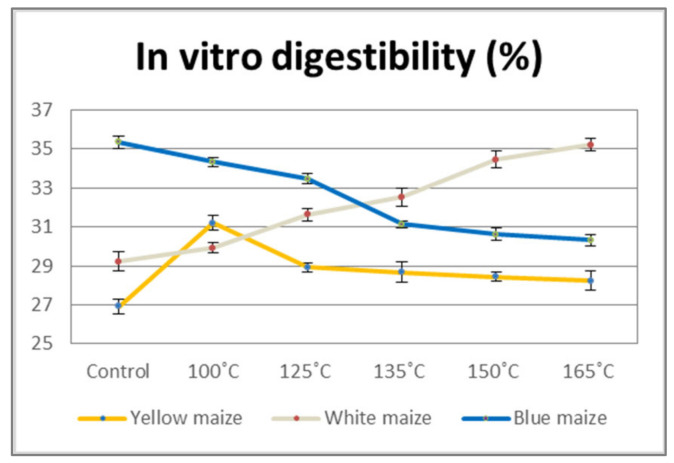
In vitro digestibility of maize flour samples.

In vitro methods were used to assess the digestibility of control and DHT maize flours. Figure 4 illustrates how the DHT significantly enhanced the in vitro digestibility of the white maize flour samples. The control sample of white maize flour had a digestibility of 29.23%, but as the temperature rose to 165 °C, the digestibility increased by 17%. As mentioned before, even though the treated and control samples’ protein contents of white maize flour were quite similar, DHT can cause structural changes in proteins and, in case of digestibility, make them more digestible by unfolding and exposing peptide bonds to digestive enzymes [61]. However, the results of blue maize flour indicated that DHT reduced digestibility, and the effect was negatively correlated with the treatment temperature. The digestibility of blue maize flour samples continuously decreased with rising temperatures, and at a temperature of 165 °C it was 14.20% lower than that of the control sample.

According to Nikolić et al. [62] these differences could be attributed to the chemical composition and physical properties of the raw material, such as kernel hardness, as well as the amylose-to-amylopectin ratio, non-starch components, and processing methods [63] that may also have an impact on the digestibility of maize flour. Additionally, Schefer et al. [64] demonstrated that phenolic compounds interact with food macromolecules, including proteins, lipids, and carbohydrates. Hence, these compounds block the digestive enzymes through chemical interactions, causing the enzymes to precipitate and decrease activity when it comes to breaking down carbs. In addition, our results demonstrated the positive correlation between phenolic compounds and in vitro digestibility (Figure 2). The in vitro digestibility of the yellow maize flour sample ranged from 26.91% to 28.44%. However, DHT at a temperature of 100 °C caused a sharp increase of 16% in digestibility compared to the control yellow maize sample. These results suggest that due to disruption/degradation of the food matrix within the maize flour at different temperatures during DHT, the bioavailability and bioaccessibility of the maize flour chemical compounds increase or decrease. Nevertheless, it should be emphasized that maize flour is often ingested following heat treatment. The in vitro digestibility in our study was determined in raw samples of maize flour as a starting point. Therefore, DHT at optimal temperature is a viable solution for pre-processing maize flour to enhance its digestibility.

### 3.6. Color of Maize Flour Samples

**Figure 5:**
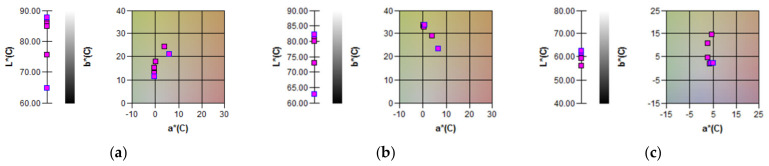
CIE a*, b*, and L* chromaticity diagram for: (**a**) white maize flour samples; (**b**) yellow maize flour samples; (**c**) blue maize flour samples.

The effect of DHT on the maize flour color parameters was evaluated, and the results are shown in Table 4 and Figure 5. The L* value corresponds to the lightness and ranged from 64.77 to 87.20, 62.80 to 82.04, and from 56.17 to 62.55 in white, yellow, and blue corn flour samples, respectively. The lightness parameter L* significantly decreased from 87.20 to about 64.77 for the control sample and DHT sample at 165 °C, respectively, indicating that the white maize flour showed darkening which was more pronounced as the DHT temperature increased. The same trend was observed for yellow and blue maize flour samples, where samples treated at a temperature of 165 °C had a lightness parameter L* of 23% and 10% smaller than that found in their control samples. Furthermore, in white maize flour, DHT caused the parameter a* to increase significantly from −0.36 for the sample treated at a temperature of 100 °C to 6.14 for the sample treated at a temperature of 165 °C. The same trend was observed for yellow maize flour samples, where the parameter a* ranged from 0.66 to 6.51 (Table 4). Given that the parameter a* is related to redness and greenness characteristics, the obtained results for the impact of DHT on blue maize flour samples were as expected. The DHT of the maize flours results in a consistent reddish hue due to the caramelization and nonenzymatic browning (Maillard reaction). However, DHT caused a significant decrease in parameter a* in the blue maize flour samples; thus, the control sample had a parameter a* 12% higher when compared to the DHT sample at 165 °C. These differences can be explained by genotypic differences in the analyzed samples, since blue popping maize has an especially high content of anthocyanins as compared to light colored maize genotypes [35,40]. The b* value of control maize flour amounted to 2.22 (blue maize), 10.12 (white maize), and 33.64 (yellow maize). These results clearly illustrate genotypic differences in the analyzed samples. Similarly to parameter a*, the parameter b* showed a significant increasing trend in white and blue maize samples with ranges from 12.99 to 21.12 and 2.15 to 14.55 for DHT samples at 100 °C and 165 °C, respectively. This rising trend suggested that the dry heat treatment enhanced the yellowness attributes, which may be due to the level of formation of the intermediate compounds during the Maillard reaction [43]. Similar results have been reported for potato and sweet potato [65], barley [66], and wheat [67]. Overall, the CIE L*a*b*color results indicated that DHT produced browning in the maize flour, as well as that the genetic basis of the material was a very important parameter in changing the color parameters.

## 4. Conclusions

In this study, we found that DHT significantly changed techno-functional properties of white, yellow, and blue maize flour samples. The obtained results revealed that DHT had great effects on the physicochemical properties of maize flour constituent structural reorganization. Additionally, DHT changed the content of bioactive compounds, chemical composition, and digestibility of investigated maize flour samples. The DHT increased the content of soluble free phenolic compounds in tested maize flour samples in comparison to those in control maize flours. DHT significantly improved the functionality of maize flour in terms of water absorption capacity, water solubility, and digestibility, thus it can be effectively used to make up for the poor functionality of raw maize flour. The white maize sample had the highest WAI and lowest WSI when applied DHT at 135 °C, which suggests that white maize flour swells more easily and could exhibit a higher viscosity. This study shows that DHT at moderate temperatures (125–135 °C) could be a viable solution for the pre-processing of maize flour to enhance its utilization potential. Future research is required given that there has been little to no prior study on the dry heat treatment of maize flour. Comprehending how the techno-functional properties of wholegrain maize flour are affected by the application of dry heat treatment and determining the ideal DHT temperature range may be advantageous and could lead to new possibilities for producing customized flours that can be used in the food sector.

## Figures and Tables

**Table 1:** Preparation of the digestion fluids.

Solution	SSF		SGF		SDF	
	pH = 7.0		pH = 3.0		pH = 7.0	
	Volume (mL)	Stock (g/L)	Volume (mL)	Stock (g/L)	Volume (mL)	Stock (g/L)
KCl	10	46.7	28.0	46.7	5.4	46.7
KH_2_PO_4_	20	68.0	0.9	68.0	0.8	68.0
NaHCO_3_	4	84.0	6.5	168.0	42.5	42.5
NaCl	1	120.0	10.0	120.0	8.0	120.0
MgCl_2_(H_2_O)_6_	1	30.0	2.0	30.0	1.1	30.0

**Table 2:** Content of protein, starch and dietary fibres in maize flour samples (% d.m.).

Sample	Temperature (°C)	Total Protein	Total Starch	NDF	ADF	ADL	Hemicellulose	Cellulose
White maize	control	8.36 ± 0.01 ^b^	71.06 ± 0.08 ^c^	11.48 ± 0.52 ^c^	3.56 ± 0.10 ^ab^	0.45 ± 0 ^b^	7.92 ± 0.62 ^c^	3.11 ± 0.10 ^a^
	100	8.34 ± 0.04 ^b^	71.07 ± 0.37 ^c^	12.35 ± 0.87 ^c^	2.67 ± 0.04 ^bc^	0.35 ± 0.09 ^b^	9.68 ± 0.91 ^c^	2.33 ± 0.13 ^ab^
	125	8.68 ± 0.06 ^a^	70.08 ± 0.11 ^d^	11.45 ± 0.58 ^c^	2.28 ± 0.01 ^c^	0.27 ± 0.04 ^b^	9.17 ± 0.57 ^c^	2.01 ± 0.03 ^b^
	135	8.35 ± 0.04 ^b^	73.76 ± 0.09 ^ab^	15.60 ± 0.58 ^b^	3.38 ± 0.45 ^ab^	0.33 ± 0.01 ^b^	12.72 ± 0.58 ^b^	3.22 ± 0.13 ^a^
	150	8.29 ± 0.06 ^b^	74.45 ± 0.08 ^a^	14.65 ± 0.10 ^b^	2.19 ± 0.08 ^c^	0.31 ± 0.06 ^b^	12.47 ± 0.18 ^b^	1.88 ± 0.14 ^b^
	165	8.24 ± 0.06 ^b^	73.34 ± 0.17 ^b^	44.35 ± 0.06 ^a^	4.02 ± 0.37 ^a^	1.62 ± 0.21 ^a^	40.34 ± 0.32 ^a^	2.40 ± 0.17 ^ab^
Yellow maize	control	12.36 ± 0.27 ^a^	69.60 ± 0.07 ^ab^	14.19 ± 0.82 ^d^	3.58 ± 0.73 ^ab^	0.40 ± 0.02 ^bc^	10.62 ± 0.09 ^d^	3.18 ± 0.07 ^a^
	100	12.20 ± 0.09 ^a^	68.43 ± 0.11 ^c^	16.79 ± 0.01 ^d^	3.98 ± 0.05 ^ab^	0.25 ± 0.01 ^c^	12.82 ± 0.04 ^c^	1.73 ± 0.06 ^a^
	125	12.31 ± 0.30 ^a^	68.58 ± 0.46 ^bc^	14.30 ± 0.20 ^d^	3.64 ± 0.45 ^ab^	0.42 ± 0.04 ^bc^	10.67 ± 0.64 ^d^	3.22 ± 0.08 ^a^
	135	12.39 ± 0.01 ^a^	69.18 ± 0.09 ^abc^	18.06 ± 0.40 ^c^	2.48 ± 0.12 ^ab^	0.30 ± 0.05 ^bc^	15.58 ± 0.28 ^c^	2.18 ± 0.07 ^a^
	150	11.97 ± 0.04 ^a^	69.61 ± 0.28 ^a^	23.88 ± 0.35 ^b^	3.10 ± 0.71 ^ab^	0.50 ± 0.09 ^b^	20.79 ± 1.07 ^b^	2.60 ± 0.08 ^a^
	165	11.99 ± 0.04 ^a^	68.37 ± 0.28 ^c^	37.84 ± 0.54 ^a^	4.41 ± 0.56 ^a^	1.13 ± 0.06 ^a^	33.43 ± 1.10 ^a^	3.28 ± 0.03 ^a^
Blue maize	control	13.00 ± 0.17 ^a^	66.82 ± 0.07 ^a^	15.59 ± 0.05 ^e^	3.77 ± 0.29 ^b^	0.54 ± 0.11 ^a^	11.82 ± 0.34 ^e^	3.23 ± 0.10 ^a^
	100	13.02 ± 0.08 ^a^	64.63 ± 0.11 ^d^	16.79 ± 0.63 ^de^	3.51 ± 0.01 ^b^	0.48 ± 0.03 ^b^	13.28 ± 0.62 ^de^	3.03 ± 0.02 ^a^
	125	13.05 ± 0.03 ^a^	64.81 ± 0.08 ^cd^	18.09 ± 0.59 ^d^	3.64 ± 0.38 ^b^	0.50 ± 0.03 ^b^	14.45 ± 0.21 ^d^	3.14 ± 0.07 ^a^
	135	13.03 ± 0.01 ^a^	65.40 ± 0.24 ^bc^	29.98 ± 0.46 ^b^	3.65 ± 0.04 ^b^	0.58 ± 0.11 ^b^	26.33 ± 0.50 ^b^	3.07 ± 0.07 ^a^
	150	12.94 ± 0.04 ^a^	67.33 ± 0 ^a^	25.42 ± 0.21 ^c^	3.87 ± 0.07 ^b^	0.85 ± 0.19 ^b^	21.55 ± 0.98 ^c^	3.02 ± 0.07 ^a^
	165	12.83 ± 0 ^a^	65.79 ± 0.25 ^b^	45.86 ± 0.36 ^a^	5.33 ± 0.35 ^a^	2.29 ± 0.45 ^b^	40.47 ± 0.80 ^a^	3.04 ± 0.10 ^a^

NDF—neutral detergent fibres, ADF—acid detergent fibres, ADL—lignin. Values are means of three determinations ± standard deviation. Means followed by the same letter within the same column and maize genotype are not significantly different according to Tukey’s test (*p* > 0.05).

**Table 3:** The content of phenolic compounds, anthocyanins and antioxidant capacity of maize flour samples (d.m. basis).

Sample	Temperature (°C)	Soluble Free Phenolics (mg GAE/kg)	Insoluble Bound Phenolics (mg GAE/kg)	Anthocyanins (mg CGE/kg)	Antioxidant Capacity (mmol trolox/kg)
White maize	control	145.89 ± 5.12 ^cd^	2181.27 ± 7.46 ^a^	n.d.	12.07 ± 2.25 ^a^
	100	131.33 ± 4.80 ^d^	1964.08 ± 15.67 ^c^	n.d.	10.05 ± 0.01 ^a^
	125	206.88 ± 2.76 ^bc^	1792.47 ± 11.99 ^d^	n.d.	10.85 ± 1.19 ^a^
	135	217.76 ± 6.04 ^b^	1817.00 ± 5.66 ^d^	n.d.	13.01 ± 0.49 ^a^
	150	251.82 ± 1.52 ^b^	2050.47 ± 12.06 ^b^	n.d.	13.04 ± 2.00 ^a^
	165	336.41 ± 8.23 ^a^	2049.65 ± 6.57 ^b^	n.d.	13.32 ± 0.29 ^a^
Yellow maize	control	300.83 ± 12.33 ^ab^	2812.76 ± 6.49 ^a^	n.d.	17.05 ± 0.98 ^a^
	100	266.76 ± 3.08 ^b^	2296.43 ± 5.77 ^d^	n.d.	14.83 ± 0.40 ^ab^
	125	312.94 ± 14.33 ^ab^	2413.24 ± 4.59 ^c^	n.d.	15.89 ± 0.14 ^b^
	135	322.33 ± 13.58 ^ab^	2745.59 ± 4.83 ^b^	n.d.	16.41 ± 0.35 ^ab^
	150	345.10 ± 7.62 ^a^	2757.16 ± 2.61 ^b^	n.d.	16.89 ± 0.13 ^a^
	165	366.56 ± 10.24 ^a^	2756.68 ± 7.72 ^b^	n.d.	17.15 ± 0.18 ^a^
Blue maize	control	390.21 ± 7.24 ^bc^	3726.57 ± 21.82 ^a^	894.62 ± 9.56 ^c^	24.03 ± 0.09 ^a^
	100	337.69 ± 7.39 ^c^	2729.09 ± 15.69 ^d^	948.78 ± 0.03 ^b^	20.51 ± 0.13 ^b^
	125	390.96 ± 2.45 ^b^	2593.54 ± 8.69 ^e^	1011.87 ± 12.79 ^a^	22.29 ± 0.39 ^ab^
	135	408.04 ± 9.74 ^b^	2601.64 ± 16.06 ^e^	927.48 ± 10.12 ^bc^	23.09 ± 1.01 ^ab^
	150	411.28 ± 11.90 ^b^	2825.41 ± 10.73 ^c^	706.37 ± 14.23 ^d^	23.74 ± 0.28 ^a^
	165	546.16 ± 3.63 ^a^	2903.46 ± 14.79 ^b^	368.72 ± 11.38 ^e^	23.96 ± 1.46 ^a^

n.d.—not detected. Values are means of three determinations ± standard deviation. Means followed by the same letter within the same column and same maize genotype are not significantly different according to Tukey’s test (*p* > 0.05).

**Table 4:** The measured CIE L*a*b*color values of the maize flour samples.

Sample	Temperature (°C)	L*	a*	b*
White maize	control	87.200 ± 0.056 ^a^	−0.390 ± 0.000 ^f^	10.130 ± 0.000 ^f^
	100	86.440 ± 0.028 ^ab^	−0.360 ± 0.000 ^e^	12.990 ± 0.013 ^e^
	125	85.690 ± 0.042 ^b^	−0.320 ± 0.000 ^d^	15.200 ± 0.000 ^d^
	135	84.800 ± 0.025 ^c^	0.130 ± 0.000 ^c^	17.770 ± 0.000 ^c^
	150	75.650 ± 0.001 ^d^	3.930 ± 0.001 ^b^	24.380 ± 0.001 ^b^
	165	64.770 ± 0.03 ^e^	6.140 ± 0.000 ^a^	21.120 ± 0.000 ^a^
Yellow maize	control	82.040 ± 0.000 ^a^	0.660 ± 0.001 ^c^	33.640 ± 0.002 ^b^
	100	81.150 ± 0.001 ^b^	0.330 ± 0.000 ^e^	33.270 ± 0.002 ^c^
	125	80.980 ± 0.001 ^c^	0.210 ± 0.002 ^f^	33.670 ± 0.002 ^a^
	135	79.900 ± 0.001 ^d^	0.630 ± 0.001 ^d^	32.790 ± 0.002 ^d^
	150	73.010 ± 0.001 ^e^	4.070 ± 0.001 ^b^	29.020 ± 0.000 ^e^
	165	62.900 ± 0.000 ^f^	6.510 ± 0.000 ^a^	23.440 ± 0.001 ^f^
Blue maize	control	62.550 ± 0.001 ^a^	5.250 ± 0.000 ^a^	2.220 ± 0.000 ^d^
	100	61.710 ± 0.002 ^b^	4.130 ± 0.000 ^c^	2.150 ± 0.001 ^e^
	125	61.490 ± 0.001 ^c^	3.570 ± 0.001 ^d^	2.000 ± 0.001 ^f^
	135	61.670 ± 0.002 ^d^	2.700 ± 0.001 ^e^	4.420 ± 0.001 ^c^
	150	59.380 ± 0.000 ^e^	2.870 ± 0.001 ^f^	10.560 ± 0.002 ^b^
	165	56.170 ± 0.000 ^f^	4.600 ± 0.001 ^b^	14.550 ± 0.000 ^a^

Means followed by the same letter within the same column and maize genotype are not significantly different according to Tukey’s test (*p* > 0.05).

## Data Availability

The original data presented in the study are openly available in RIK at https://rik.mrizp.rs/?locale-attribute=sr_RS; https://nutribreedinghub.rs/outcomes/publications/ accessed on 5 September 2024.
